# A Novel Memristive Multilayer Feedforward Small-World Neural Network with Its Applications in PID Control

**DOI:** 10.1155/2014/394828

**Published:** 2014-08-14

**Authors:** Zhekang Dong, Shukai Duan, Xiaofang Hu, Lidan Wang, Hai Li

**Affiliations:** ^1^School of Electronics and Information Engineering, Southwest University, Chongqing 400715, China; ^2^Department of MBE, City University of Hong Kong, Hong Kong; ^3^Department of ECE, University of Pittsburgh, Pittsburgh, PA 15261, USA

## Abstract

In this paper, we present an implementation scheme of memristor-based multilayer feedforward small-world neural network (MFSNN) inspirited by the lack of the hardware realization of the MFSNN on account of the need of a large number of electronic neurons and synapses. More specially, a mathematical closed-form charge-governed memristor model is presented with derivation procedures and the corresponding Simulink model is presented, which is an essential block for realizing the memristive synapse and the activation function in electronic neurons. Furthermore, we investigate a more intelligent memristive PID controller by incorporating the proposed MFSNN into intelligent PID control based on the advantages of the memristive MFSNN on computation speed and accuracy. Finally, numerical simulations have demonstrated the effectiveness of the proposed scheme.

## 1. Introduction

In 1971, Professor Chua theoretically formulated and defined the memristor and described that the memristance (short for resistor of a memristor) is characterized by the relationship between the electrical charge *q* and flux *φ* passing through a device [1]. However, it was only after the first physical realization of the memristor in nanoscale at Hewlett-Packard (HP) Lab in 2008 that it immediately garnered extensive interests among numerous researchers [2–4]. The reported experiments confirmed that the memristor possesses switching characteristic, memory capacity, and continuous input and output property. Due to these unique properties, memristors are being explored for many potential applications in the areas of nonvolatile memory [5, 6], very-large-scale integrated (VLSI) circuit [7], artificial neural networks [8–10], digital image processing [11–13], and signal processing and pattern recognition [14]. At present, a considerable number of models of different complexity have been proposed in the literatures, such as Pickett's model [15], spintronic memristor model [16], nonlinear ionic drift model [17], boundary condition-based model [18], and threshold adaptive memristor model [19]. These published models exhibit desired nonlinearity of nanoscale structures. This paper still applies the TiO_2_ memristor model on account of its simplified expressions and the same ideal physical behaviors.

Brain neural network emerges from the interactions of dozens, perhaps hundreds, of brain regions, each containing millions of neurons [20]. They are highly evolved nervous systems capable of high-speed information processing, real-time integration of information across segregated sensory channels, and brain regions [20, 21]. In order to obtain the similar intelligence of human brain, artificial neural network is designed to imitate the human brain not merely on architecture but also on work patterns. The connection structure of artificial neural networks is generally divided into feedforward, feedback, single-layer, multilayer, and so forth. Most of these connection architectures are approximately regular. However, the bioneurological researches show that brain neural network has random features to a certain degree and exhibits “small-world” effectiveness, that is, high levels of clustering and short average path length [22]. Therefore, it becomes a hot issue to design bionics neural network with randomness in architecture based on the background of neurobiology.

Notably, Watts and Strogatz revealed a significant effect that is in common among complex networks. They pointed out that the real architecture of network is nearly a middle model between regular connection and random connection and defined it as small-world network (WS model) in 1998 [23]. Over the past several years, a large number of investigations on complex networks have provided new insight into biological neural networks. Bassett concluded that human brain functional networks have small-world network topology derived from a series of magneto encephalography experiments [22]. Douw et al. found that the cognition is related to the resting-state small-world network topology [24]. In literature [25], the authors applied small-world properties into prefrontal cortex that correlate with predictors of psychopathology risk, which holds promise as a potential neurodiagnostic for young children. Taylor has studied the protein structures and binding based on small-world network strategies and has made great progress [26]. Simard built up a small-world neural network through rewiring the regular connections and found that the small-world neural network has faster learning speed and smaller error than that of the regular network and random network with the same size [27]. In this paper, we incorporate the memristor into the multilayer feedforward small-world neural network to build up a new type of memristive neural network that is easy of VLSI implementation and closer to biological networks. Furthermore, based on the proposed memristive neural network, a novel memristive intelligent PID controller is put forward. The nanoscale memristor is beneficial for easily adjusting the PID control parameters and the hardware realization of modern intelligent microcontrol system.

This paper is organized as follows. In Section 2, we derive the mathematical model of a nonlinear memristor which takes into account the nonlinear dopant drift effect nearby the terminals and the boundary conditions and give its Simulink model correspondingly. Following that, the concepts and design algorithm of the memristive small-world neural network are described in detail in Section 3.  Section 4 designs a memristive PID controller by combining the proposed neural network with the standard PID control theory. In order to guarantee the feasibility and effectiveness of the proposed scheme, the computer simulations are performed in Section 5. Finally, we give the conclusions in Section 6.

## 2. The Nonlinear Memristor Model

### 2.1. The Mathematical Model of the Memristor

**Figure 1:**
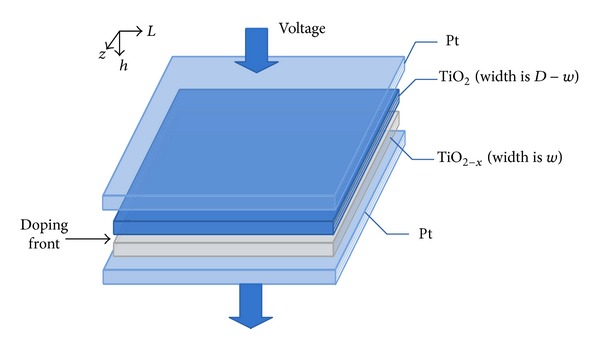
Schematic model of the HP memristor.

A memristor or memristive device is essentially a two-terminal passive electronic element with memory capacity. Its memristance state depends on the amplitude, polarity, and duration of the external applied power. The physical model of the HP memristor from [28], shown in Figure 1, consists of a two-layer thin film (thickness *D* ≈ 10 nm) of TiO_2_ sandwiched between two platinum electrodes. One of the layers, which is described as TiO_2−*x*_, is doped with oxygen vacancies (called dopants) and thus it exhibits high conductivity. The width *w* of the doped region is modulated depending on the amount of electric charge passing through the memristor. The other TiO_2_ layer owning an insulating property has a perfect 2 : 1 oxygen-to-titanium ratio, and this layer is referred to the undoped region. Generally, an external excitation *v*(*t*) applied across the memristor may cause the charged dopants to drift and the boundary between the two regions would be moved correspondingly with the total memristance changed eventually.

The total resistance of the memristor, *M*, is a sum of the resistances of the doped and undoped regions:

(1) $$\begin{array}{c} M\left(t\right)=R_{\mathrm{on}}\left(\frac{w\left(t\right)}{D}\right)+R_{\mathrm{off}}\left(1-\frac{w\left(t\right)}{D}\right), \end{array}$$

where *R*_*on*⁡_ and *R*_*off*⁡_ are the limited values of the memristance for *w* = *D* and *w* = 0, respectively. Setting the internal state variable as *x* = *w*/*D*, ∈[0,1], (1) can be rewritten as

(2) $$\begin{array}{c} M\left(t\right)=R_{\mathrm{off}}+\left(R_{\mathrm{on}}-R_{\mathrm{off}}\right)x\left(t\right). \end{array}$$

When *t* = 0, the initial memristance is

(3) $$\begin{array}{c} M_{0}=R_{\mathrm{off}}+\left(R_{\mathrm{on}}-R_{\mathrm{off}}\right)x_{0}. \end{array}$$

The movement speed of the boundary between the doped and undoped regions depends on the resistance of doped area, the passing current, and other factors according to the state equation:

(4) $$\begin{array}{c} \frac{dx}{dt}=ki\left(t\right)f\left(x\right),\;\;k=\frac{\mu_{v}R_{\text{ON}}}{D^{2}}, \end{array}$$

where *μ*_*ν*_ ≈ 10^−14^ m^2^ s^−1^ V^−1^ is the average ionic mobility parameter. As we all known, small voltages can yield enormous electric fields in nanoscale devices, which can secondarily produce significant nonlinearities in the ionic transport. As for a memristive device, these nonlinearities are manifested particularly at the thin film edges, especially at the two boundaries. This phenomenon, called nonlinear dopant drift, can be simulated by multiplying a proper window function *f*(*x*) on the right side of (4). Based on [28], there are several kinds of classical window functions, such as Joglekar window function and Biolek window function. This paper chooses the Joglekar window function which can be described by

(5) $$\begin{array}{c} f\left(x\right)=1-{\left(2x-1\right)}^{2P}, \end{array}$$

where *P* is a positive integer called the control parameter.

**Figure 2:**
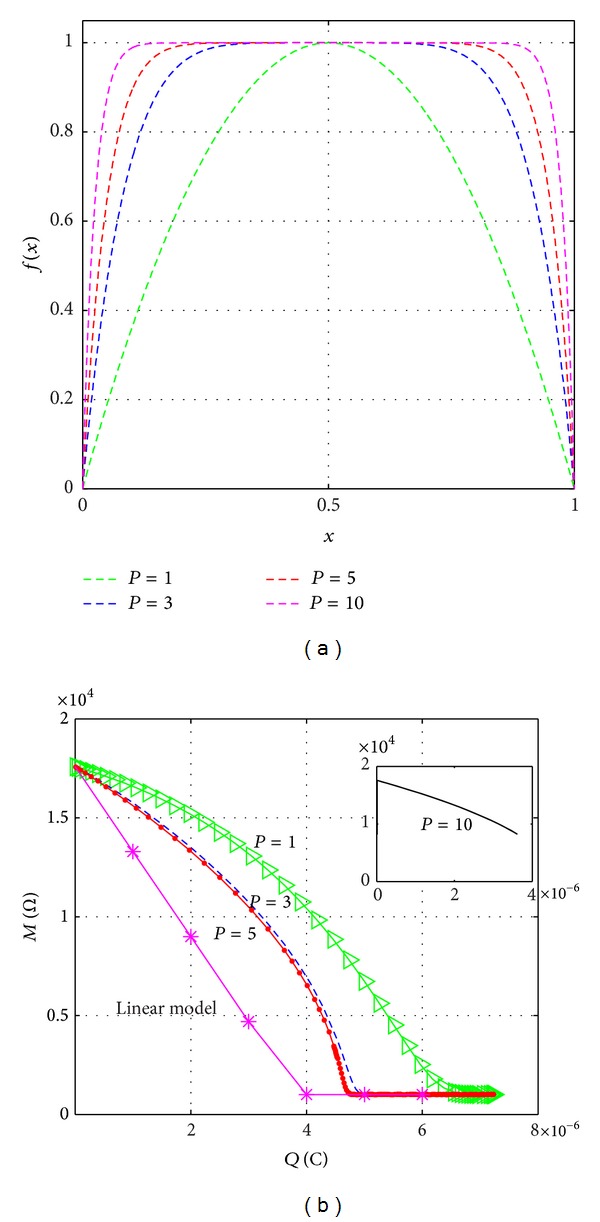
The influence of different values of the integer *P* on the memristor. (a) Joglekar window function for *P* = 1, *P* = 3, *P* = 5, and *P* = 10. (b) Relationship between memristance versus charge for the nonlinear memristor model. As the integer *P* increases, the graphs tend to linearity.

Figure 2(a) exhibits the behavior of the Joglekar window function for different values of *P*.  Figure 2(b) shows the graphs of the memristance versus charge of the memristor. As the value of *P* becomes smaller, the nonlinearity increases. On the other hand, as the integer *P* increases, the model tends to the linear model. Based upon this, as well as the literature [3, 28], we set the value of the integer *P* = 1 in this window function and obtain

(6) $$\begin{array}{c} f\left(x\right)=4x-4x^{2}. \end{array}$$

Substituting (6) into (4),

(7) $$\begin{array}{c} \overset{x(t)}{\underset{x_{0}}{\int }}\left(\frac{1}{x\left(\tau \right)}+\frac{1}{1-x\left(\tau \right)}\right)dx\left(\tau \right)=\overset{t}{\underset{0}{\int }}4ki\left(\tau \right)d\tau , \end{array}$$

where the internal state variable satisfies *x*(*τ*) ∈ [*x*_0_, *x*(*t*)] and the integration time is 0 ≤ *τ* ≤ *t*.

Assume *q*_0_ = 0; we can get

(8) $$\begin{array}{c} \frac{x\left(t\right)}{1-x\left(t\right)}=\frac{x_{0}}{1-x_{0}}\times e^{4kq(t)}. \end{array}$$

The initial value of the state variable can be expressed as

(9) $$\begin{array}{c} x_{0}=\frac{R_{\mathrm{off}}-R_{0}}{\Delta R}. \end{array}$$

Then, the expression of *x*(*t*) can be calculated as

(10) $$\begin{array}{c} x\left(t\right)=1-\frac{1}{Ae^{4kq(t)}+1}, \end{array}$$

where *A* is a constant and its value is determined by *R*_*off*⁡_, *R*_*on*⁡_, and *R*_0_:

(11) $$\begin{array}{c} A=\frac{R_{\mathrm{off}}-R_{0}}{R_{0}-R_{\mathrm{on}}}. \end{array}$$

Combining (2)–(11), the resistance of memristor can be rewritten as

(12) $$\begin{array}{c} M\left(t\right)=R_{\mathrm{on}}+\Delta R\frac{1}{Ae^{4kq(t)}+1}, \end{array}$$

where Δ*R* = *R*_*off*⁡_ − *R*_*on*⁡_.

**Figure 3:**
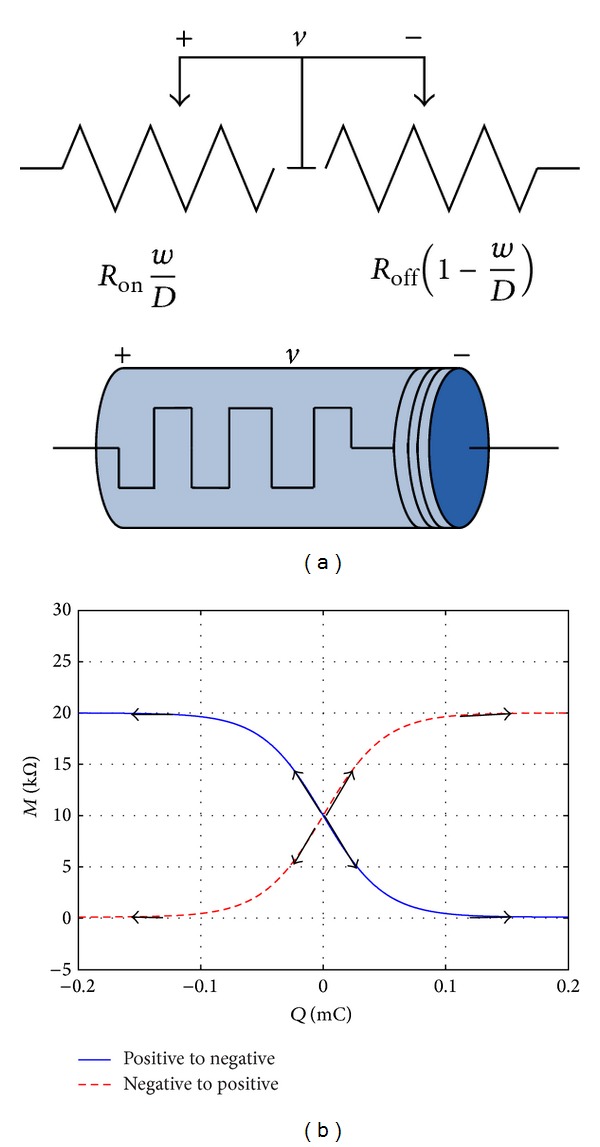
The nonlinear memristor model and its characteristic curves. (a) The equivalent circuit of the memristor and its 3D symbol. (b) The relationship between memristance and the charge.

Giving a sine stimulus to the memristor, we get the simulation results using MATLAB software. It is noteworthy that the memristor is a two-terminal element with polarity, which is shown in Figure 3(a). When the current flows into the memristive device from the positive pole to the negative pole, one can get the relationship curve (the blue line) between memristance and charge through it as shown in Figure 3(b). On the contrary, when the current flows into the memristor from the negative pole to the positive pole, the relationship curve is denoted by the red dashed line. When the charge is close to or exceeds the charge threshold values, the resistance of the memristor reaches and stays at *R*_*on*⁡_ and *R*_*off*⁡_, respectively. Notably, the threshold value denotes the quantity of electric charge required when the memristance reaches the limit resistance. The parameters of the model are *R*_*on*⁡_ = 100 Ω, *R*_*off*⁡_ = 20 kΩ, *M*_0_ = 10 kΩ, *D* = 10 nm, and *μ*_*ν*_ ≈ 10^−14^ m^2^s^−1^V^−1^. Moreover, the simulation results in Figure 3(b) are consistent with the results concluded by Adhikari et al. in [8, 29].

### 2.2. The Simulink Model of the Memristor

**Figure 4:**
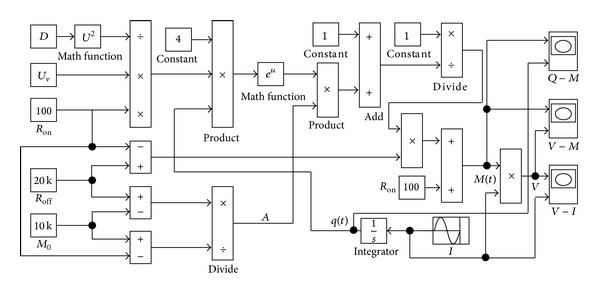
The Simulink model of the nonlinear memristor.

For the sake of analyzing the characteristics of the memristor model comprehensively, a Simulink model is built upon (2)–(12) and illustrated in Figure 4. The model mainly consists of input and output modules, internal operation modules (multipliers, adders, and modules), and parameter control modules. The model parameters are the same as those in Figure 3. The signal stimulus applied into the memristor is a sinusoidal current source with amplitude of 0.5 mA and frequency of 1 Hz.

**Figure 5:**
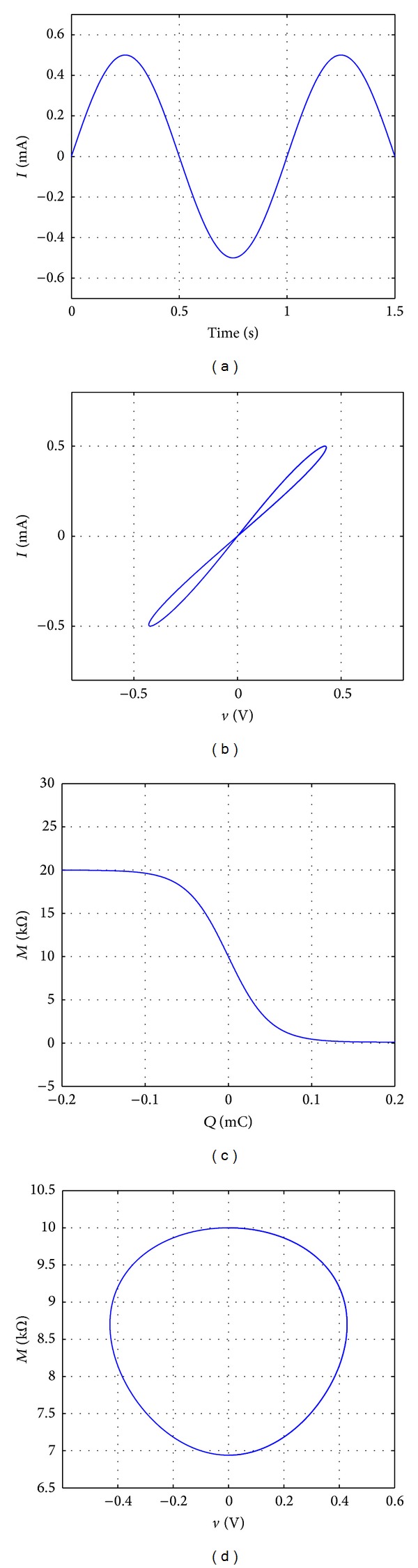
The results of the memristor Simulink model. (a) The input current source. (b) Relationship between the current *i* and the voltage *v*. (c) Relationship between the memristance *M* and the charge *q*. (d) Relationship between the memristance *M* and the voltage *v*.

The simulation results are exhibited in Figure 5. The current flowing through the memristor is shown in Figure 5(a). The typical hysteresis loop in Figure 5(b) shows its switching characteristic; that is, the memristance can switch between high resistance and low resistance.  Figure 5(c) illustrates that the memristance is a nonlinear function of the flow of charge as discussed previously.  Figure 5(d) shows the relationship between the memristance *M* and the charge *q*. Notably, in the part of the higher memristance state, the change ratio of the memristance is low, while, in the part of the lower memristance state, the change ratio of the memristance is high.

## 3. The Memristive Multilayer Feedforward Small-World Neural Network

### 3.1. The Multilayer Feedforward Small-World Neural Network

Generally, small-world phenomenon indicates that a network has highly concentrated local connections and also includes a few random long connections. In real world, a large number of networks have the small-world effect, such as disease transmission network, social network, and the food chain network [22]. As is known to all, in the classical multilayer feedforward neural network, such as BP network, the *i*th neuron in the *l*th layer *v*_*i*_^*l*^ only connects its neighboring neuron sets *V* ^*l*−1^ and *V* ^*l*+1^. In addition, all connections are feedforward and no connections exist between neurons within the same layer. This kind of network can be considered as a regular network. Based on [27] and the construction process of WS small world model, we introduce Algorithm 1 which is used to construct multilayer feedforward neural network model according to the rewiring probability. The specific construction process is given as follows.

*Step  1.* Initialization: assuming the number of the network layers is *L*, each layer has *n*_*l*_ neuron nodes and the rewiring probability is *P*. 

**Figure 6:**
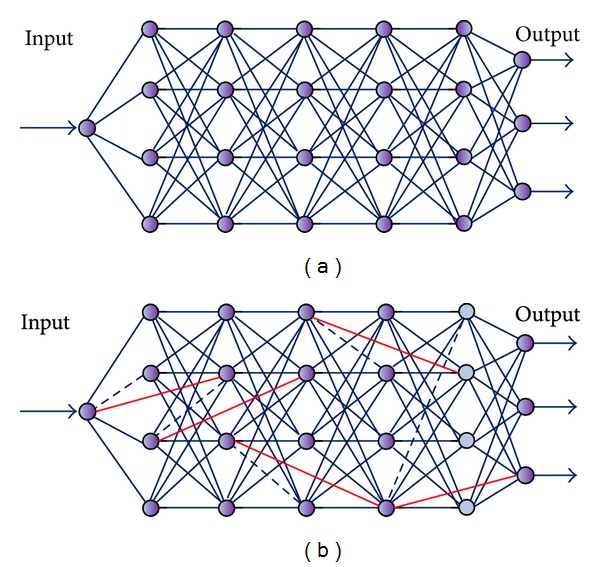
The construction progress of the multilayer feedforward WS small-world neural network. (a) The regular network (*P* = 0). (b) The multilayer feedforward WS small-world neural network (0 < *P* < 1).

*Step  2.* Generate the multilayer feedforward regular neural network, as shown in Figure 6(a). 

*Step  3.* As shown in Algorithm 1 where *p*_1_(*h*) is the probability to select reconnection layer, selection probability between two neurons decreases exponentially. *α* and *β* are the distance coefficients, rand and randint both are MATLAB functions, the former is used to generate a number between (0,1) randomly, and the latter can be used to randomly generate an integer from 1 to *n*_*l*_. Since the connections of the (*L* − 1)th layer cannot generate new long-connections if they are disconnected, the connections of the last two layers are not reconnected in the network.

As shown in Figure 6(a), when the rewiring probability *P* = 0, the connection of the network maintains completely regular mode. Nonetheless, when *P* ranges from 0 to 1, the long cross-layer connections are generated according to the rewiring probability *P* and the probability of reconnection layer selecting. The resulting structure is between completely regular and random connection mode, as shown in Figure 6(b).

More specially, we set the network connection matrix as *W*, where *W* ^*l*^denotes the connection submatrix between the *l*th layer and the (*l* + 1)th layer. *w*_*ij*_^*l*^ ∈ *R* is the connection weight between the neuron *i* of the *l*th layer and the neuron *j* of the (*l* + 1)th layer. If there exists connection between these two neurons, then *w*_*ij*_^*l*^ ≠ 0; otherwise, *w*_*ij*_^*l*^ = 0. Therefore, the regular network connection matrix can be expressed as the following equation:

(13) $$\begin{array}{c} W=\left(\begin{array}{ccccccc} 0 & W^{1} & 0 & 0 & 0 & 0 & 0\\ 0 & 0 & W^{2} & 0 & 0 & 0 & 0\\ 0 & 0 & 0 & W^{3} & 0 & 0 & 0\\ 0 & 0 & 0 & 0 & W^{4} & 0 & 0\\ 0 & 0 & 0 & 0 & 0 & W^{5} & 0\\ 0 & 0 & 0 & 0 & 0 & 0 & W^{6}\\ 0 & 0 & 0 & 0 & 0 & 0 & 0 \end{array}\right), \end{array}$$

in which the number zero means no connection exists between the corresponding layers. As for multilayer feedforward small-world neural network, because of the reconnection performance, the connection matrix changes into as

(14) $$\begin{array}{c} W^{\prime }=\left(\begin{array}{ccccccc} 0 & W^{1} & B_{1}^{3} & B_{1}^{4} & B_{1}^{5} & B_{1}^{6} & B_{1}^{7}\\ 0 & 0 & W^{2} & B_{2}^{4} & B_{2}^{5} & B_{2}^{6} & B_{2}^{7}\\ 0 & 0 & 0 & W^{3} & B_{3}^{5} & B_{3}^{6} & B_{3}^{7}\\ 0 & 0 & 0 & 0 & W^{4} & B_{4}^{6} & B_{4}^{7}\\ 0 & 0 & 0 & 0 & 0 & W^{5} & B_{5}^{7}\\ 0 & 0 & 0 & 0 & 0 & 0 & W^{6}\\ 0 & 0 & 0 & 0 & 0 & 0 & 0 \end{array}\right), \end{array}$$

where *W* ^*l*^ represents the reconnection submatrix between the *l*th layer and the (*l* + 1)th layer, and the *B*_*l*_^*l*′^ is the submatrix between two nonadjacent layers, which *l* ∈ {1,2,…5}, *l*′ ∈ {3,4,…7}.

### 3.2. The Combination of the MFSNN and the Memristor

#### 3.2.1. The Memristive Synapse

The nanoscale memristor has high potential of information storage on account of the non-volatility with respect to long periods of power-down, so it can be used as electric synapse in the artificial neural networks, and the primary reasons are manifold. Firstly, as a kind of analog component, this device can realize weight updating continuously. Moreover, the memristor possesses the capacity of information storage due to the nonvolatility. This feature is consistent with the memory ability of the neurons in human's brain. Additionally, the memristive neural network can be further integrated in crossbar array which has significant advantages in better information processing capacity and huger storage.

According to the nonlinear memristor model in Section 2, the memristive conductance can be calculated from (12) as

(15) $$\begin{array}{c} G\left(t\right)=\frac{1}{M\left(t\right)}=\frac{1}{R_{\mathrm{on}}+\Delta R\left(1/\left(Ae^{4kq(t)}+1\right)\right)}. \end{array}$$

**Figure 7:**
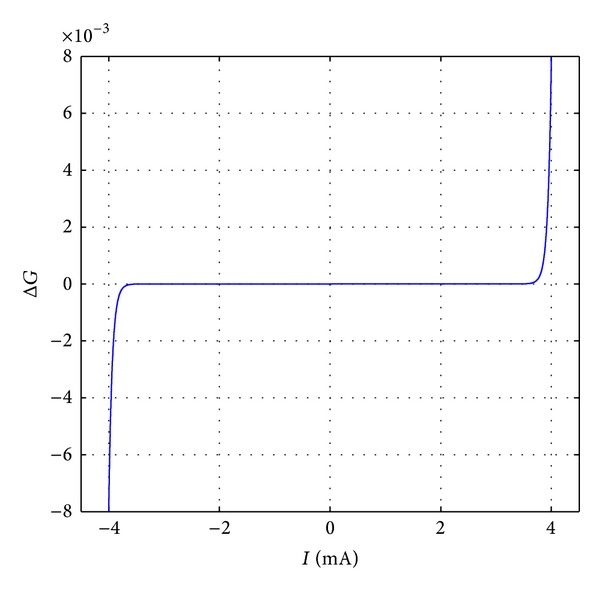
The relationship curve between the rate of the memristive conductance change and the current.

Differentiating (15) with respect to time *t*, we can be obtain

(16) $$\begin{array}{c} \frac{dG\left(t\right)}{dt}=\frac{4kAe^{4kq(t)}\Delta R}{{\left(R_{\mathrm{on}}Ae^{4kq(t)}+R_{\mathrm{off}}\right)}^{2}}\times \frac{dq\left(t\right)}{dt}, \end{array}$$

where the current *i*(*t*) = *dq*(*t*)/*dt*. Notably, when Δ*t* → 0, *dG*(*t*) ≈ Δ*G*. Hence, the rate of the memristive conductance Δ*G* can be described as the synapse weight update rule. The relationship curve between the rate of the memristive conductance change and the current is shown in Figure 7. When the current is tiny, the memristive conductance is almost invariant. While the current tends to ±4 mA, the memristive conductance changes suddenly. So the current threshold value of the memristive synapse can be set as |*I*_th_| = 4 mA.

#### 3.2.2. The Memristive Activation Function

In the standard MFSNN, the activation function for each neuron is usually the nonlinear Sigmoid function. Particularly, the activation function of the hidden layer adopts bipolar Sigmoid function, but the output layer activation function is unipolar Sigmoid function.

**Figure 8:**
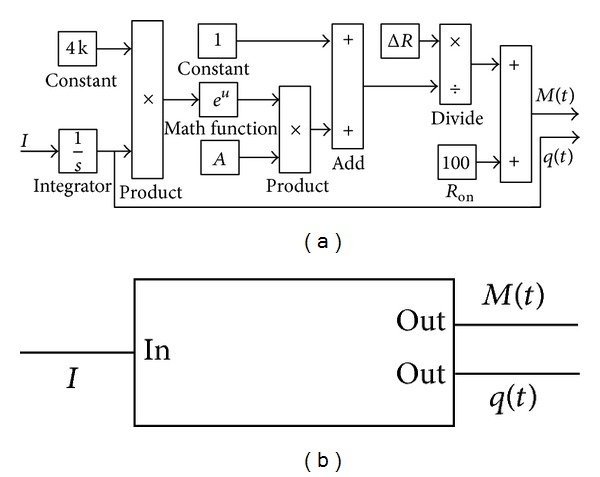
The simplified memristor model. (a) The simplified Simulink model of the memristor. (b) The package of the memristor.

Based on the constitutive relationship of the memristor, a lot of nonlinear curves can be simulated and substituted [5, 6, 11, 12]. Based on the Simulink model of the nonlinear memristor described in Section 2, we get its simplified Simulink model accordingly as shown in Figure 8(a). Furthermore, we design a package of the memristive device (in Figure 8(b)) which can be considered as a system with single-input and double-output. In this system, the input variable is the current *I*, and the output variable is the memristance *M*(*t*) and charge *q*(*t*), respectively.

Then, the behavior of the output curve can be adjusted efficiently by the parameter control module, gain module, and internal operation module, which is crucial to implement the activation function in the neural network. Here, we set the activation functions for the hidden layer and output layer neurons of the memristive MFSNN as *h*(*x*) and *y*(*x*), respectively.

**Figure 9:**
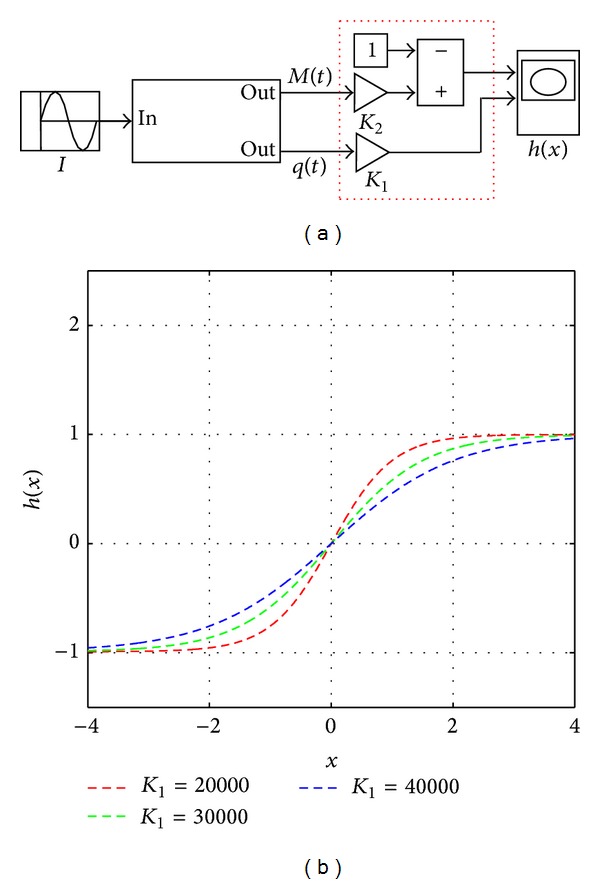
The principle diagram of the memristive activation function in the hidden layer. (a) The Simulink model of the memristive activation function in the hidden layer. (b) The curve of the memristive activation function *h*(*x*).

Figure 9(a) exhibits the constructing principle diagram of the memristive activation function in the hidden layer, in which the red dotted line frame represents the parameter adjustment area. *K*_1_ is the adjustable gain which is used for controlling the shape of the activation function, and *K*_2_ is the fixed gain whose value is *K*_2_ = 10^−4^. The suitable parameters of the memristor are chosen as *R*_*on*⁡_ = 100 Ω, *R*_*off*⁡_ = 20 kΩ, *M*_0_ = 10 kΩ, *D* = 10 nm, and *μ*_*ν*_ ≈ 10^−14^m^2^s^−1^V^−1^. The input signal is a sinusoidal current with an amplitude of 0.5 mA and a frequency of 1 Hz. Notably, the polarity of the voltage applied into the memristor is opposite to the polarity of the memristor itself; that is, the current flows through the memristor from the negative polar to the positive polar.  Figure 9(b) shows the memristive activation function of the hidden layer, and its shape varies with different values of *K*_1_.

**Figure 10:**
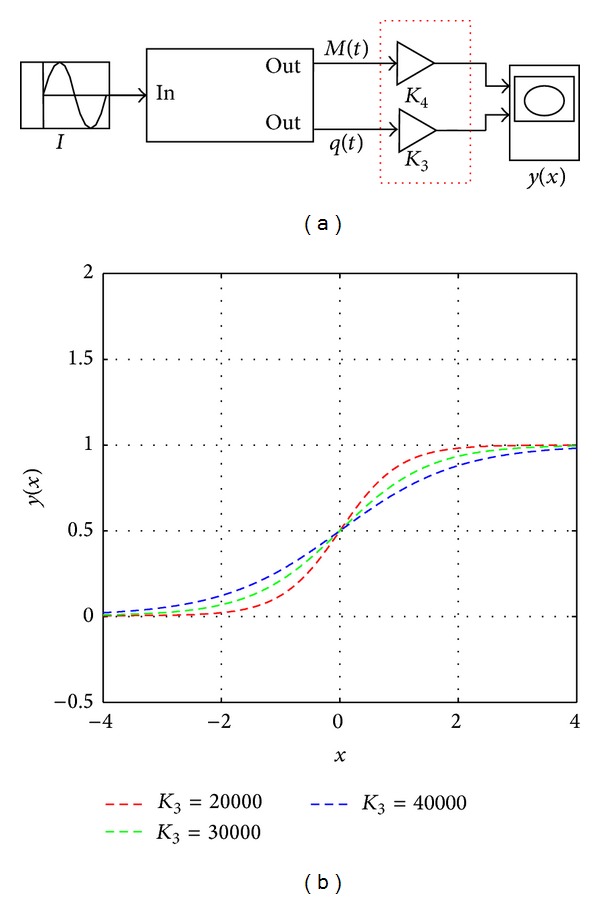
The principle diagram of the memristive activation function of the output layer. (a) The Simulink model of the memristive activation function of the output layer. (b) The curve of the memristive activation function *y*(*x*).

Similarly, Figure 10(a) is the constructing principle diagram of the activation function of the output layer. In the parameter adjustment part (the red dotted line frame), *K*_3_ is an adjustable gain and *K*_4_ is the fixed gain whose value is *K*_4_ = 2 × 10^−4^. The parameters are the same with the simulation in Figure 9.  Figure 10(b) shows the memristive activation function of the output layer. Obviously, as the value of *K*_3_ increases, the graphs tend to flatten.

## 4. The Memristive Intelligent PID Controller

So far, the PID control has found widespread applications in the modern control field. By adjusting the control action of the proportion, integration, and differentiation, we get an interactive nonlinear relationship among these control variables. The neural network has the ability of expressing the nonlinearity, which can be used in the PID control for implementing the optimal nonlinear relationship among control variables. In this work, we build up a more intelligent PID controller with the parameters (*k*_*p*_, *k*_*i*_, and *k*_*d*_) self-tune based on the presented memristive multilayer feedforward small-world neural network.

According to the literature [30], the classical incremental digital PID control algorithm can be described as

(17) u(k)=u(k−1)+kp(e(k)−e(k−1)) +kie(k)+kd(e(k)−2e(k−1)+e(k−2)),

where the *k*_*p*_, *k*_*i*_, and *k*_*d*_ are the coefficient of the proportion, integration, and differentiation, respectively.

**Figure 11:**
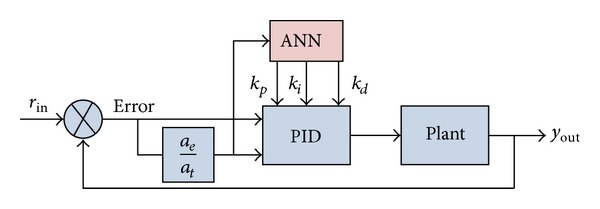
The structure diagram of the memristive neural network PID controller.

In Figure 11, the ANN is the memristive multilayer feedforward small-world neural network. Its learning algorithm consisted of the backward error propagation and the forward input signal propagation. Different from the traditional multilayer feedforward neural network, the state of the neurons in each layer not only affects the state of the neurons in the next layer but also affects the state of the neurons in the cross-layer.

Based on the novel neural network presented in Section 3, we set the *j*, *i*, and *l* that represent the input layer, hidden layer, and output layer, respectively. The number of the input layer is 1 which is same with that of the output layer, and the number of the hidden layer is *S*. *x*_*i*_ represents the input vector of the network, then the set of the input samples is *O*_*j*_^1^ = [*x*_1_, *x*_2_, *x*_3_,…, *x*_*N*_]. The number of the input vectors is dependent on the complexity of the system. Notably, the superscript 1 represents the first layer in the whole neural network.

The input and output vectors of the first hidden layer can be expressed as

(18) netii1(k)=∑j=0Nwiji1Oj1Oii1(k)=h(netii1(k))i=1,2,3,…,Q,

where the superscript *i*_1_ denotes the first hidden layer of the network and *h*(*x*) is the memristive bipolar sigmoid function proposed in Section 3.

By that analogy, the input and output vectors of the *S*th hidden layer can be written as

(19) netiis(k)=∑j=0NwijisOj1+∑a=1s−1∑b=1swiaisisnetbis(k)Oiis(k)=h(netiis(k))i=1,2,3,…,Q.

Finally, the input and output vectors of the output layer can be obtained as

(20) netls+2(k)=∑j=0Nwils+2Oj1+∑a=1s∑b=1swials+2netbs+2(k)Ols+2(k)=y(netls+2(k))l=1,2,3,

where *y*(*x*) denotes the memristive unipolar sigmoid function. The three nodes of the output layer are corresponding with the nonnegative adjustable parameters *k*_*p*_, *k*_*i*_, and *k*_*d*_ of the PID controller, respectively.

From [27], we conclude the weight update algorithm of the memristive multilayer feedforward small-world neural network as below:

(21) $$\begin{array}{c} \Delta w_{li}^{s+2}\left(k\right)=\alpha \Delta w_{li}^{s+2}\left(k-1\right)+\eta \delta_{l}^{s+2}O_{i}^{s+1}\left(k\right)\\ w_{li}^{s+2}\left(k+1\right)=w_{li}^{s+2}\left(k\right)+\Delta w_{li}^{s+2}\left(k\right), \end{array}$$

where *α* is the inertial coefficient, whose scope ranges from 0 to 1, and *η* ∈ (0,1) is the learning rate.

## 5. Computer Simulations and Results

In this section, some numerical simulations of the memristive multilayer feedforward small-world neural network PID controller have been executed on MATLAB software. The mathematical model of the controlled plant is given as

(22) $$\begin{array}{c} y_{\text{out}}\left(k\right)=\frac{a\left(k\right)y_{\text{out}}\left(k-1\right)}{1+y_{\text{out}}^{2}\left(k-1\right)}+u\left(k-1\right), \end{array}$$

where the *a*(*k*) is slow time-variant and its expression is *a*(*k*) = 1.2(1 − 0.8*e* ^0.1*k*^).

The memristive neural network under investigation is constituted by seven layers with four neurons in the input layer, three neurons in the output layer, and five in each of the five hidden layers. The learning rate of the network *η* = 0.4, and the inertial coefficient *α* = 0.05. The initial weighs as random values fall in [−0.5 0.5], and the value of the rewiring probability is chosen as *P* = 0, *P* = 0.08, *P* = 0.1, and *P* = 0.2, respectively. The parameters are *R*_*on*⁡_ = 100 Ω, *R*_*off*⁡_ = 20 kΩ, *M*_0_ = 10 kΩ, *D* = 10 nm, and *μ*_*ν*_ ≈ 10^−14^ m^2^s^−1^V^−1^, *K*_1_ and *K*_3_ are user-specified parameters whose value both are 20000, and the action time is *ts* = 0.001 s. When the system works steadily, the tracking results can be gotten as follows.

**Figure 12:**
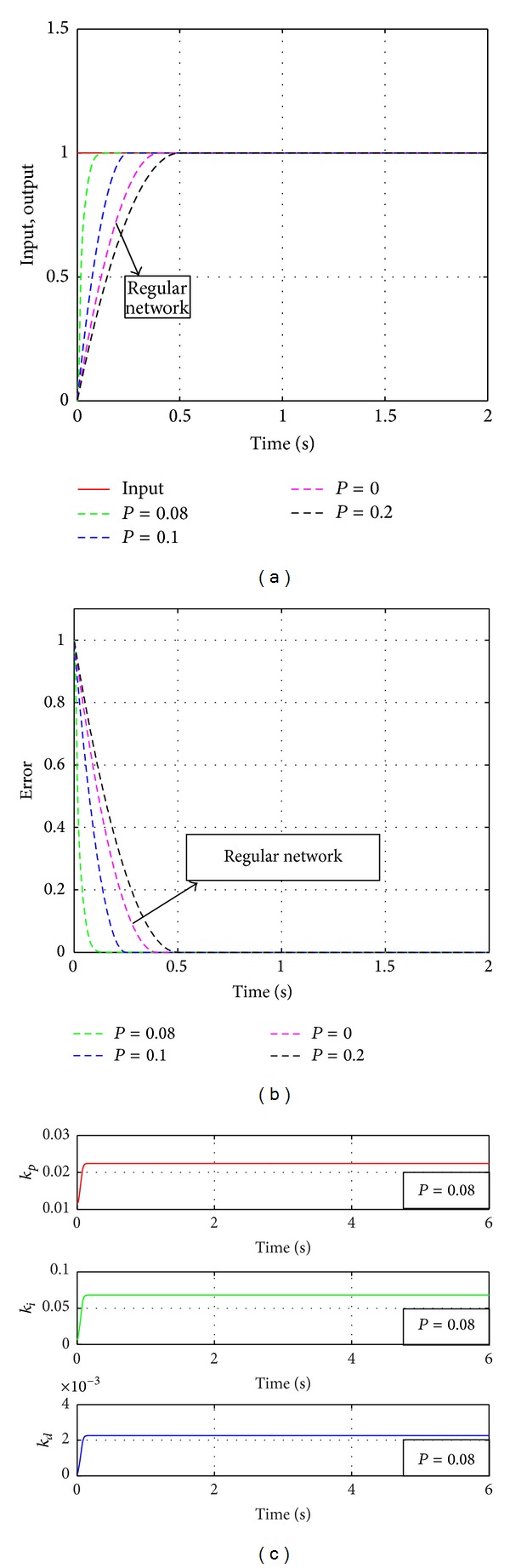
The simulation results of the memristive neural network PID controller (*r*_in_(*k*) = 1.0) under a different rewiring probability *P*. (a) The step response curve. (b) The error curves. (c) The curves of the control parameters when the rewiring probability *P* = 0.08.

Figure 12(a) shows the input signal (step response curve *r*_in_(*k*) = 1.0) and the output curves under a different rewiring probability *P*. As can be seen from the figure, when the time *t* = 0.5 s, the whole system reaches the steady state. Making a further analysis, we can conclude that when the rewiring probability *P* = 0, the memristive neural network keeps regularly in architecture. Its respond speed is slower than that of network when the rewiring probability *P* = 0.08 and *P* = 0.1. Moreover, Figure 12(b) exhibits the error curves between the input signal and the output signal correspondingly. When the rewiring probability *P* = 0.08 and *P* = 0.1, the network spends less time on approaching the predefined approximation error than the regular network (when *P* = 0).  Figure 12(c) shows the output variables of the memristive multilayer feedforward small-world neural network when *P* = 0.08 which are the control parameters *k*_*p*_, *k*_*i*_, and *k*_*d*_, correspondingly.

**Figure 13:**
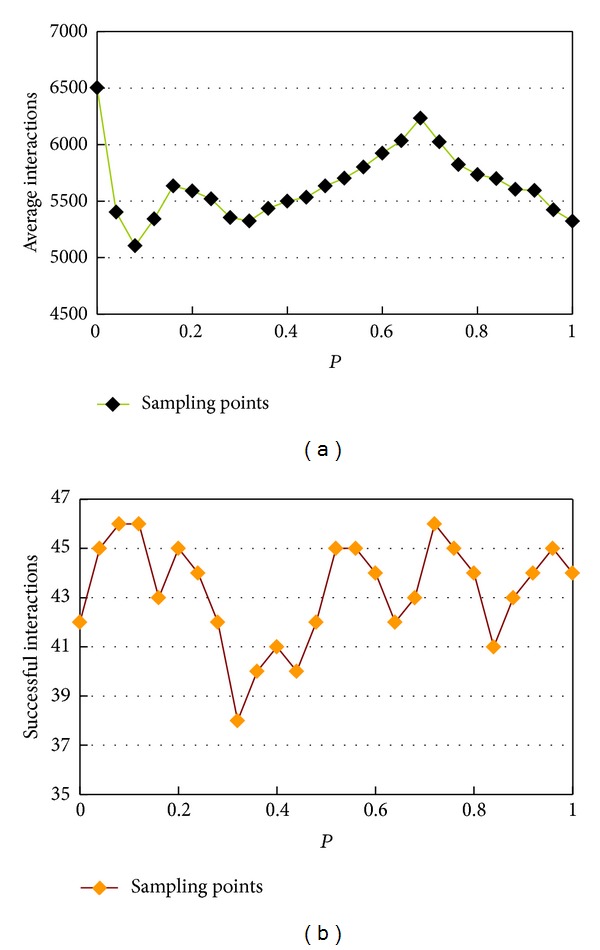
The convergence performance of the memristive neural network under different *P*. (a) The relationship between iteration and rewiring probability. (b) The effective approximation number in 50 times simulations under varying rewiring probability.

In order to verify the superior performance of the memristive small-world neuronal networks and figure out the optimal structure, we conducted a series of simulations to observe the convergence performance of the proposed network under different *P*.  Figure 13(a) shows the approximation speed (iteration times) of different network structures, that is, the smallest iteration number for reaching the predefined approximation error *ɛ* = 0.0001. Each drawn point is the average value of 50 times runs. It can be observed that the small-world networks (0 < *P* < 1) need much less iteration times than the regular neural network (when *P* = 0), which demonstrates its advantage in processing speed. Furthermore, when *P* = 0.08, the network has the fast approximation speed.

Notably, the mathematical function of this system has the local minimum, for getting out of the local minimum, we define the maximum allowable iteration times to be 10000, as previously mentioned for each *P*, and we performed the simulation for 50 times, where the effective approximation times, that is, error <0.0001 within 10000 iterations, are presented in Figure 13(b). It can be found that the small-world networks have higher accuracy rate than the regular network.

## 6. Conclusions

A mathematical closed-form charge-governed memristor model is recalled firstly and the corresponding Simulink model is presented. Using the change rule of memconductance, a memristive realization scheme for synaptic weight is proposed. Moreover, the activation functions in electric neurons are also implemented based on the single-input and double-output package of the memristor. Combining the proposed memristive synapse and activation functions, a memristor-based MFSNN is addressed. It exhibits advantages in computation speed and accuracy over the traditional multilayer neural networks by considering the small-world effect. Meanwhile, it has potential of hardware realization of the neural network because of the nanoscale size of the memristive synapse. These superior properties can further improve the application of the neural networks, such as in the intelligent controller design. Motivated by this, we apply the memristor-based MFSNN to classical PID control, and the proposed memristive PID controller may possess the following superiorities. (i) Its nanoscale physical implementation could promote the development of the microcontroller. (ii) Because of the participation of the memristive neural network, the proposed PID controller can realize the parameters self-adjustment. (iii) The control speed and accuracy are improved. Eventually, extensive numerical simulations justify the effectiveness and efficiency of the memristive PID controller over the regular neural network PID controller. This work may provide a theoretical reference to physically realize the small-world neural networks and further promote the development of modern intelligent control technology.

## Figures and Tables

**Algorithm 1:**
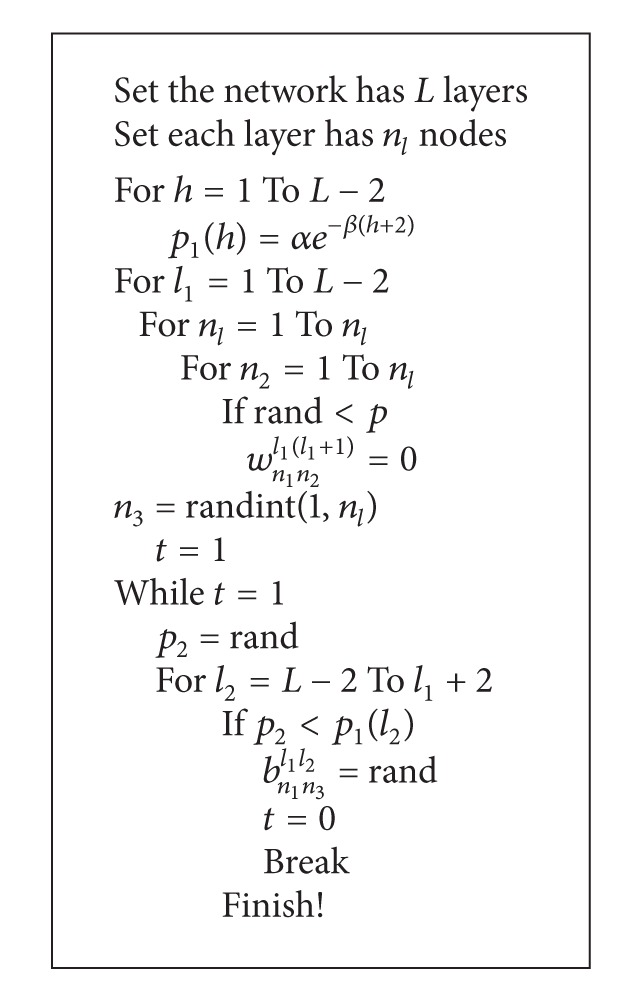
Constructive procedure of the small-world neural network.
